# Toward Memory in a DNA Brush: Site-Specific Recombination Responsive to Polymer Density, Orientation, and Conformation

**DOI:** 10.1021/jacs.3c01375

**Published:** 2023-04-18

**Authors:** Noa Avidan, Michael Levy, Shirley S. Daube, Roy H. Bar-Ziv

**Affiliations:** Department of Chemical and Biological Physics, The Weizmann Institute of Science, Rehovot 7610001, Israel

## Abstract

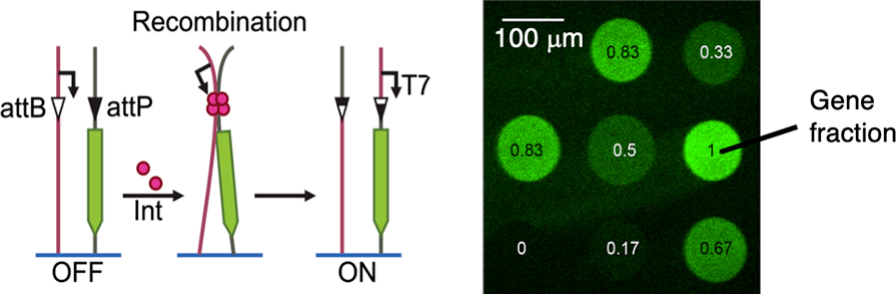

Site-specific recombination is a cellular process for the integration, inversion, and excision of DNA segments that could be tailored for memory transactions in artificial cells. Here, we demonstrate the compartmentalization of cascaded gene expression reactions in a DNA brush, starting from the cell-free synthesis of a unidirectional recombinase that exchanges information between two DNA molecules, leading to gene expression turn-on/turn-off. We show that recombination yield in the DNA brush was responsive to gene composition, density, and orientation, with kinetics faster than in a homogeneous dilute bulk solution reaction. Recombination yield scaled with a power law greater than 1 with respect to the fraction of recombining DNA polymers in a dense brush. The exponent approached either 1 or 2, depending on the intermolecular distance in the brush and the position of the recombination site along the DNA contour length, suggesting that a restricted-reach effect between the recombination sites governs the recombination yield. We further demonstrate the ability to encode the DNA recombinase in the same DNA brush with its substrate constructs, enabling multiple spatially resolved orthogonal recombination transactions within a common reaction volume. Our results highlight the DNA brush as a favorable compartment to study DNA recombination, with unique properties for encoding autonomous memory transactions in DNA-based artificial cells.

## Introduction

The implementation of complex functions such as reading and writing to memory in minimal cell-free systems using basic biological building blocks is an important step on the way to the construction of artificial cell systems. Inspired by living cells that utilize regulatory circuits and cell memory to perform complex calculations on a variety of inputs, reconstructing such processes outside of a cellular context would allow us to construct de novo artificial systems capable of performing biochemical computation.

Site-specific DNA recombination has long been recognized as a synthetic memory tool to stably embed and store information in DNA.^1^ Viral enzymes that perform site-specific DNA recombination have been used in vivo to create logical functions and alter cell states in a variety of organisms,^2−5^ and several orthogonal recombinases have been characterized in *Escherichia coli*,^6^ with a recent systematic expansion of the recombinase repertoire displaying increased sequence diversity and improved functional properties.^7^ DNA recombination has also been characterized and used as a tool in vitro, mainly for high-throughput cloning purposes,^8,9^ and autonomous DNA editing based on cell-free expression of CRISPR nucleases in an *E. coli* extract has been recently demonstrated.^10^ DNA recombination has much to offer as a simple realization of logic and memory, requiring few extra factors in autonomous cell-free systems, yet has not been implemented in such a system so far.

The immobilization of linear double-stranded DNA polymers on the surface of a biochip as DNA brushes provides a unique synthetic scenario to study recombination reactions. At high densities close to that found in vivo, DNA brushes localize the gene expression machinery against entropic macromolecular exclusion^11,12^ and provide highly crowded and confined conditions that promote intermolecular interactions crucial for multicomponent biomachine assembly.^13,14^ Unique to DNA brushes, the alignment of sequences within the brush leads to the directionality of gene expression reactions, which remarkably affects the mRNA and protein synthesis yields.^15,16^ Thus, DNA brushes may provide favorable conditions for DNA recombination reactions involving interactions between a recombinase and two DNA substrates and enable investigation of these reactions under conditions that better mimic the crowded and restricted motion of DNA in cells.^17^ What’s more, DNA brushes have been implemented in synthetic artificial cell models to form geometrically controlled spatial and temporal patterns, such as propagation of gene expression^18^ and genetic oscillators.^19^ Toward implementation of DNA-based memory transactions in these systems, here, we demonstrate a cascaded reaction of cell-free transcription–translation in a minimal reconstituted system (PURE)^20^ of Bxb1 integrase, a well-characterized and efficient unidirectional serine recombinase,^21,22^ that, once expressed, localizes to the brush and mediates sequence-specific gene switching.

We first show that the unidirectional DNA strand exchange reaction between two recombinase-specific DNA sites in the DNA brush was faster than a similar reaction in bulk solution. The recombination yield was dependent on the ratio between the two DNA substrates, reaching about 50% for a 1:1 ratio, and almost full recombination when one substrate was in excess over the other. Unique to a DNA brush scenario, we further show that the recombination yield was dependent non-linearly on the interpolymer distance and on the proximity of the recombination site to the surface anchoring point: the exponent of the power law dependence on gene fraction approached 2 for recombination sites very close to the surface and approached 1 for lengths much larger than the interpolymer distance. Finally, we show that localization of the reaction to the DNA brush allows us to carry out parallel recombination reactions with a single recombinase within the same reaction volume, opening possibilities for versatile DNA-based computations in a minimal system.

## Results

### Site-Specific Recombination Kinetics and Yield in a DNA Brush

**Figure 1:**
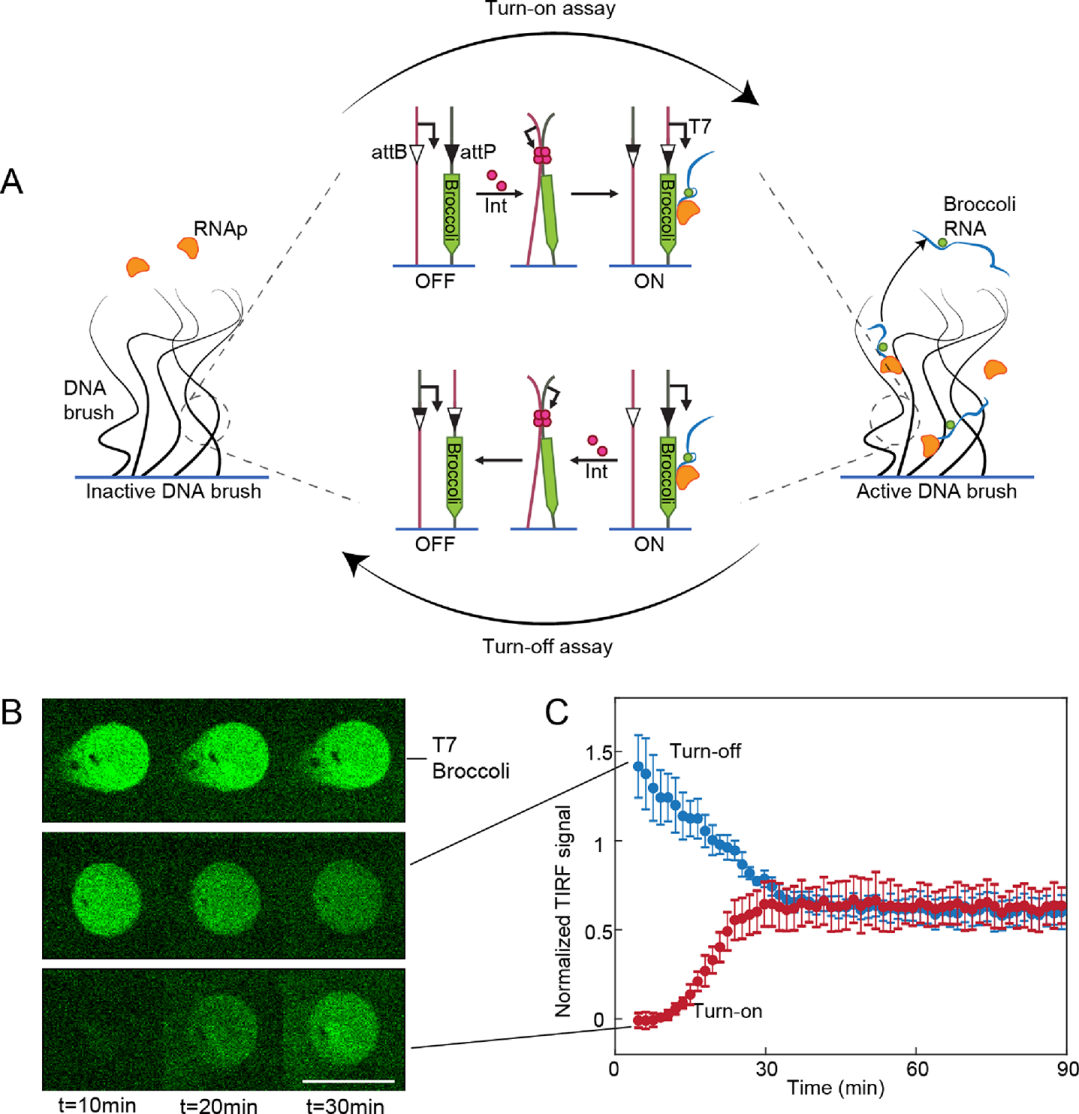
Transcription-based site-specific recombination in DNA brushes. (A) Schemes of regulation of transcription in a DNA brush via recombination by Bxb1 integrase (pink). Top: Recombination between attB (white triangle) and attP (black triangle) sequences on adjacent DNA strands transfers a T7 promoter (arrow) upstream of a long gene encoding a broccoli aptamer. Transcription by T7 RNA polymerase (orange) is turned on, and the fluorescent broccoli-RNA molecules accumulate inside the brush. Bottom: Recombination separates between the gene and the promoter, turning transcription off. (B) Time lapse of TIRF images of three DNA brushes encoding broccoli-modified RNA. Top: Steady transcription (no att sequences). Middle: Turn-off assay. Bottom: Turn-on assay. Scale bar: 100 μm. (C) Kinetics of the normalized broccoli turn-on and turn-off assays measured in the DNA brush averaged over five repeats and normalized by a brush with steady transcription. The non-normalized data are shown in Figure S2.

We designed an intermolecular turn-on assay based on Bxb1 integrase^21,22^ in which a recombination reaction within a DNA brush turns on the expression of a reporter gene (Figure 1A). The attP and attB sequences,^23^ which are the recognition sites for integrase binding, cleavage, and ligation, were positioned upstream to the coding sequence of a promoter-less reporter gene on one DNA construct and downstream to a promoter on another, respectively, thereby dictating the directionality of the recombination reaction (Supporting Information and Table S1). A complete recombination reaction should merge the promoter and the gene in the correct orientation to yield reporter gene synthesis. In addition, we designed a turn-off assay in which the attP sequence is set between a promoter and a reporter gene. Recombination with a construct containing only attB should separate the promoter from the reporter gene, interrupting the expression.

To directly measure the recombination kinetics, we embedded a fluorescent broccoli aptamer sequence^24^ in a gene coding for a long RNA as a reporter of transcription. We applied a PURE in vitro transcription–translation system,^20^ supplemented with a plasmid coding for Bxb1 integrase (Supporting Information), onto surfaces patterned with DNA brushes. The DNA brushes were composed of the reporter and promoter DNA constructs in either the turn on or turn off assay formats, all enclosed in a PDMS chamber of 3 mm in diameter and a height of about 100 μm^14^ (Supporting Information). Using TIRF microscopy, we detected a fluorescent signal of the broccoli aptamer appearing localized to the DNA brushes (Figure 1B). The fluorescent signal was found to increase linearly with the fraction of active promoters in the initial state of the brush (Figure S1), suggesting that the fluorescent signal corresponded to the number of active promoters that recombined with the reporter gene. Therefore, transcription was a result of a reaction cascade, starting with integrase expression in the solution, followed by integrase-mediated DNA recombination, which turned the synthesis of the broccoli aptamer either on or off depending on the constructs’ design.

We measured the dynamics of RNA synthesis in a DNA brush consisting of the turn-off assay genes at a 1:1 ratio. RNA synthesis levels were reduced over a ∼20 min period and then stabilized after a 60% reduction in the signal, suggesting that the recombination process saturated at this point with 60% efficiency. The decrease in signal of the turn-off assay corresponded with the increase in signal measured in an equivalent turn-on assay (Figure 1B,C), supporting the notion that the RNA signal reflected the dynamics of the recombination process. Note that a turn-on assay is not suitable to measure the recombination yield as it does not define the maximal recombination level that could be reached. Only a turn-off assay, which starts with maximal expression that is then turned off by recombination, could quantitatively evaluate the recombination yield. Overall, these results demonstrated that on-chip bulk expression of integrase led to a functional enzyme that could overcome brush exclusion, search and bind the att sites within the dense brush, and perform recombination between two immobilized DNA polymers.

**Figure 2:**
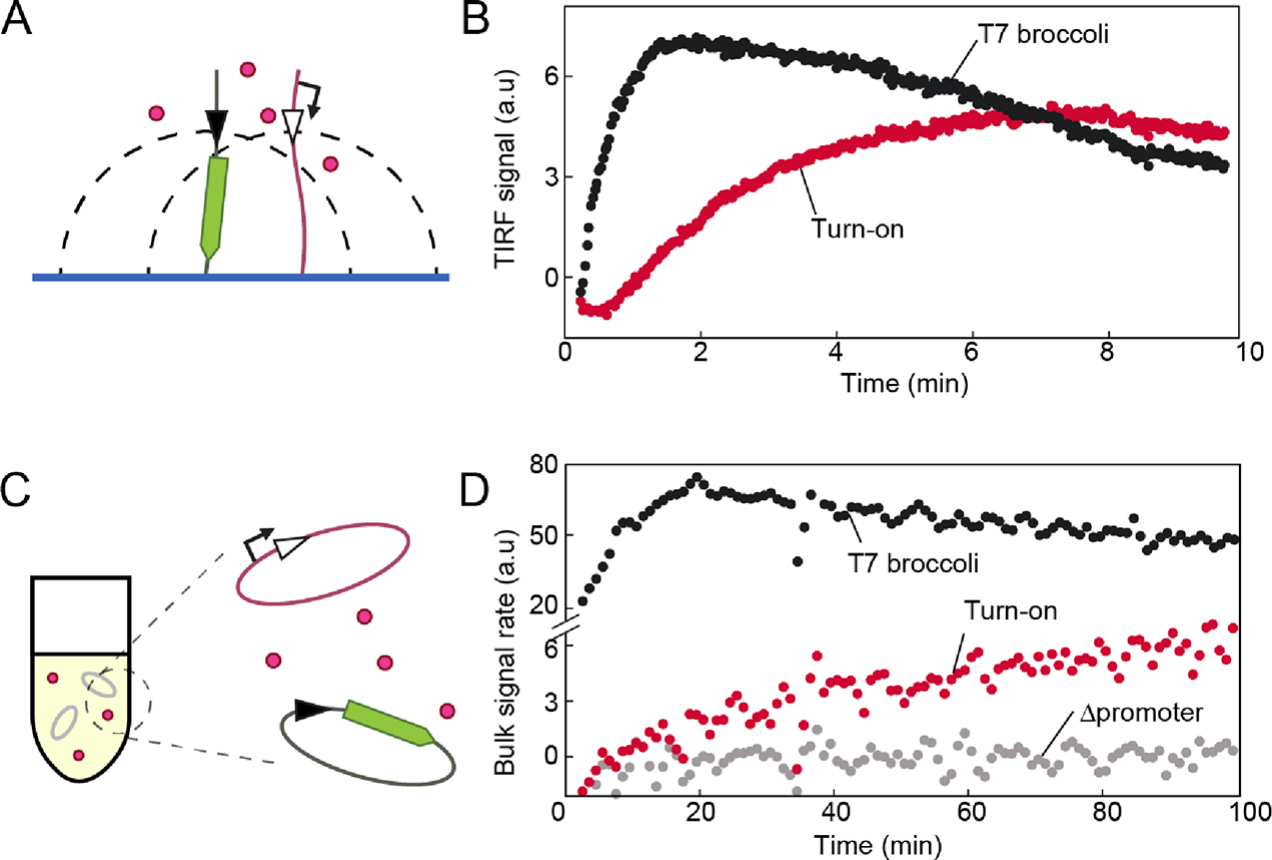
Net recombination dynamics in DNA brushes and in bulk reactions. (A, B) Scheme and dynamics of a turn-on broccoli transcription assay (as in Figure 1A, top) in a DNA brush driven by pre-expressed Bxb1 integrase (Supporting Information). The DNA containing the att sites is immobilized on the surface, which limits its movement to a volume represented by the dashed line. The TIRF signals, averaged over three repeats and background-subtracted, are the actual rates of steady broccoli transcription (black) and recombination-dependent transcription (red) as no RNA is accumulating in the TIRF measurements. (C, D) Scheme and transcription rate of a turn-on broccoli transcription assay in a bulk reaction with pre-expressed Bxb1 integrase and the two DNA constructs as plasmids. Rate was calculated as the fitted slope of four points centered on the timepoint of measurement. The measured fluorescence curve is shown in Figure S3 for comparison.

We asked whether the ∼20 min period in which the fluorescent signal changed and stabilized (Figure 1C) reflected only the dynamics of the recombination reaction or, in addition, the dynamics of integrase expression. To distinguish between the two, we pre-expressed integrase in the PURE expression system prior to adding it to DNA brushes containing the turn-on assay (Supporting Information) and began measurement immediately upon its addition. The recombination reaction reached its half point after ∼3 min compared to a brush with steady broccoli transcription (Figure 2A,B). We then compared this net recombination time in a DNA brush to the net recombination time off-chip between two plasmids using pre-expressed integrase added to a PURE reaction (Supporting Information) (Figure 2C). Because the TIRF broccoli signal measures the net change between local RNA synthesis and diffusion away from the brush, whereas the bulk broccoli measures the total accumulation of RNA, we took the time derivative of the bulk signal (Figure 2D). The bulk experiment half-point of the fluorescent broccoli signal was reached in ∼30 min (Figure 2D and Figure S3).

Since it is difficult to compare the concentration of the DNA substrates between the two scenarios, we can qualitatively conclude that recombination in the brush was faster than between free-floating plasmids in solution. This is likely due to the favorable conditions in the brush that reduce the effective volume that must be explored by the integrase-bound att sites due to the sites being closer together on average in the brush, similarly to the search mechanisms at reduced dimensionality suggested for DNA-binding proteins.^25^ The brush density is ∼1000 DNA polymers/μm^2^,^16^ and the average height of the free end of the DNA polymer random coil above the surface is ∼100 nm.^26^ Therefore, the DNA brush has an effective site concentration of about 10 μM, three orders of magnitude above the DNA concentration in the bulk experiments, which was 3 nM of each plasmid. This would considerably increase the probability of the recombination sites to find each other and recombine. The recombination reaction is an intermolecular DNA–DNA polymer interaction mediated by the integrase, which due to DNA immobilization is effectively an intramolecular interaction. After one recombination event is performed, the integrase may remain in the region of the DNA brush, possibly making following recombination reactions even faster.

**Figure 3:**
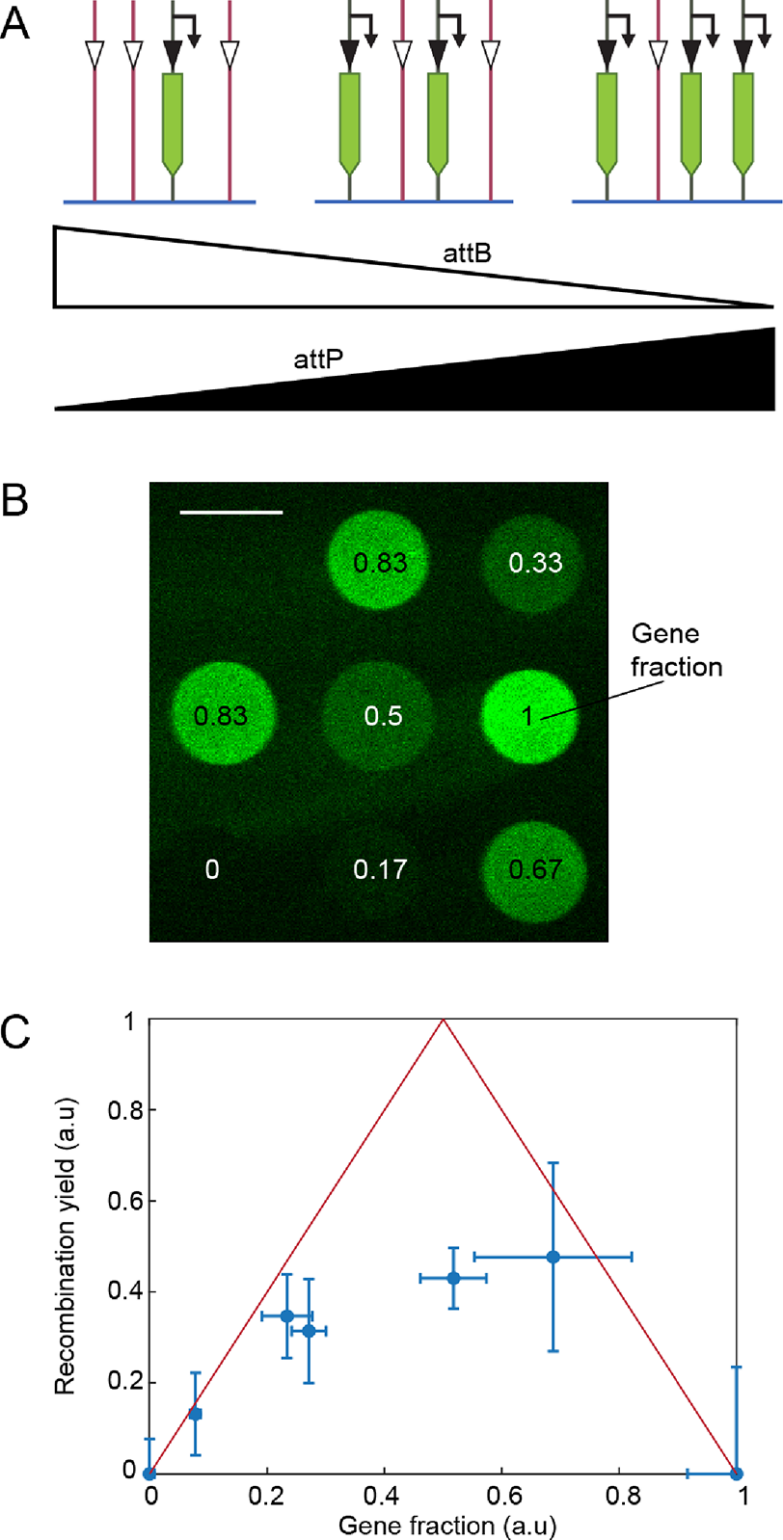
Recombination yield in DNA brushes. (A) Experimental scheme to measure recombination yield in DNA brushes containing the broccoli-based turn-off assay at different ratios between the gene and turn-off constructs. Color scheme as in Figure 1. Integrase was expressed from a plasmid added to the bulk. (B) TIRF image of a well containing the DNA brushes with different ratios between the turn-off constructs at the beginning of the experiment. The fraction of the broccoli gene out of the entire brush is indicated for each brush. Scale bar: 100 μm. (C) Yield of recombined gene fraction calculated from the data in (B) plotted as a function of the initial gene fraction and averaged over 15 repeats for each DNA type. The kinetics of this experiment are shown in Figure S4. The maximal possible yield, assuming the recombination reaction completely depletes one of the reactants, is shown in red.

We noted that recombination in the DNA brush at a 1:1 ratio between the reporter gene and the turn-off DNA gave only half of the expected yield (Figure 1C). We wondered whether we could improve the yield by changing the ratios, which would effectively cause one construct to be in excess, allowing the other to recombine with a higher probability. We immobilized DNA brushes, each with a different ratio between recombination constructs and all in the same reaction volume, and measured the RNA synthesis in DNA brushes (Figure 3A,B). In Figure 3C, we show the recombination yield defined as the fraction of the brush consisting of DNA polymers that have undergone recombination, which is calculated as twice the difference between the broccoli signal at the beginning and the end of the kinetics divided by the signal in a full broccoli-transcribing brush. In an ideal brush with the two DNA constructs perfectly mixed and organized in a 2D lattice, the yield should approach 1 and drop linearly with gene fractions smaller and larger than 0.5 (Figure 3C, red curve). The experimental yield deviated from the ideal brush around gene fraction 0.5 (a 1:1 gene ratio) with a ∼50% recombination yield. The yield was much closer to the maximal possible value when one DNA construct was in excess over the other. This suggests that the deviations may stem from an inhomogeneous distribution of the two types of DNA constructs. If one construct is surrounded by its duplicates, no recombination could occur even though there are more available constructs to recombine with. We can then drive the reaction by adding the other construct in excess.

### Effect of Inter-DNA-Polymer Distance on Recombination

We asked whether the efficiency of recombination was responsive to the average distance between gene constructs in the brush. DNA brushes provide a simple means to vary interpolymer distance by diluting the recombination constructs with noncoding DNA at a fixed total DNA density. Due to dilution, we had to develop a recombination assay with increased sensitivity as the broccoli signal diffuses away from the surface upon gene transcription. We developed an alternative translation-based assay that relied on trapping the fluorophores on the surface, increasing the fluorescent signal.

**Figure 4:**
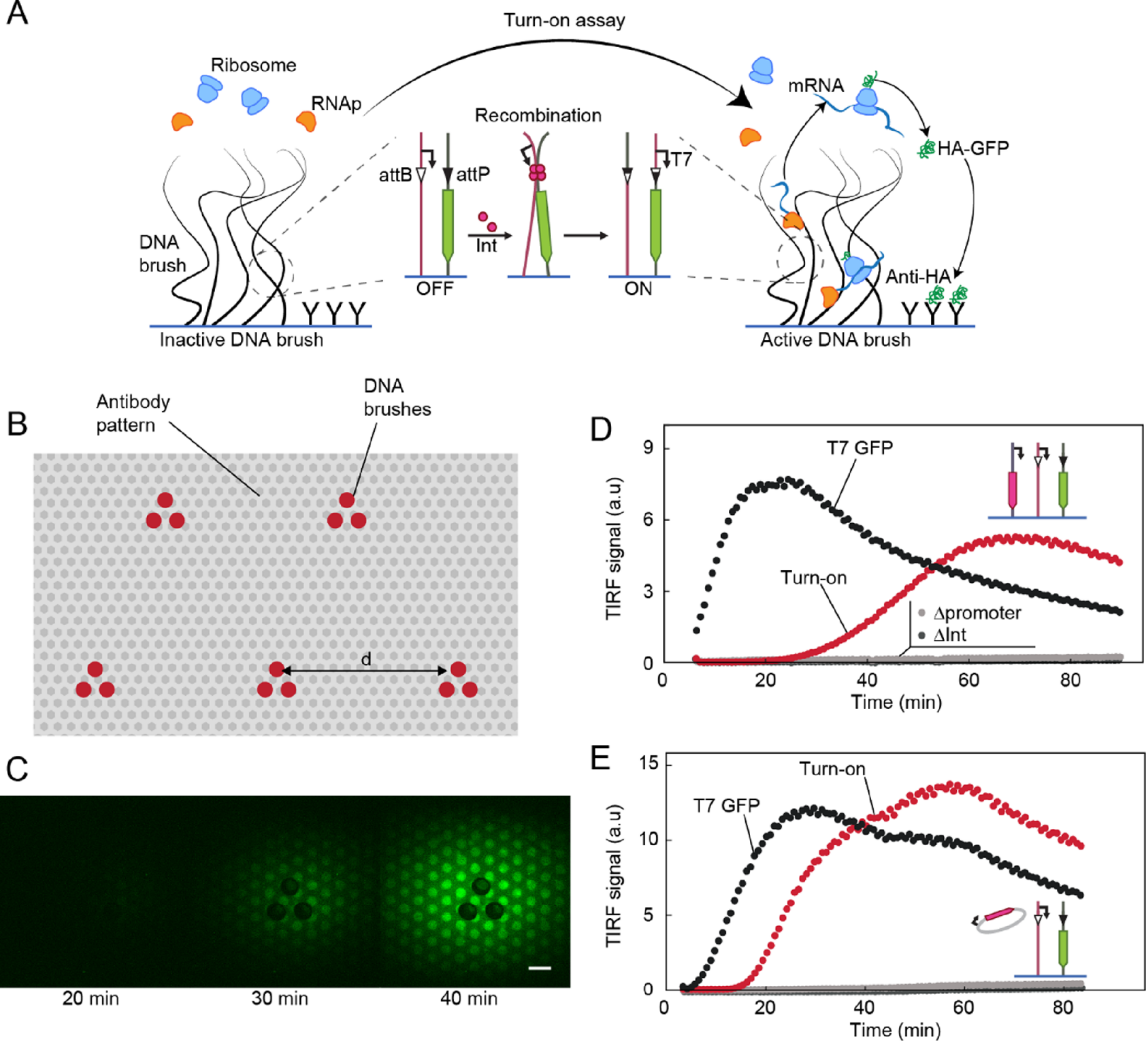
Sensitive recombination turn-on assay of GFP expression and surface capture. (A) Scheme of GFP expression mediated by Bxb1 integrase recombination between a promoter upstream of an attB site (white triangle) and an HA-GFP gene (light green) downstream of an attP site (black triangle). HA-GFP (dark green) expressed by T7 RNA polymerase (orange) and ribosomes (blue) is trapped on surface-immobilized anti-HA antibodies (Supporting Information). (B) Scheme of antibody hexagonal pattern (gray) surrounding DNA brushes (red) spotted on the surface as a three-brush cluster. The distance *d* between two brush clusters within the same well is greater than 1 mm in order to prevent cross-talk. (C) TIRF images of three time points of GFP expression and surface trapping in a well patterned as in (B). Scale bar: 100 μm. (D) HA-GFP trapping kinetics independent of recombination (black) or mediated by the integrase (red) expressed from the brush along with the promoter and HA-GFP constructs at a ratio of two integrase genes per one of each substrate gene. (E) HA-GFP trapping kinetics as in (D) except that the integrase was expressed from a plasmid added to the bulk. Repeats of (D) and (E) are shown in Figures S6 and S7, respectively.

The alternative assay was a turn-on assay based on merging of a promoter on one DNA construct to a green fluorescent protein (GFP) gene on another DNA construct in an orientation that yields GFP synthesis (Figure 4A). Recombinase-mediated turn-on of GFP expression has been demonstrated before by flipping the promoter or the gene sequence^27^ rather than by merging two DNA constructs. The GFP was tagged with HA peptide for surface capturing, enabling high-sensitivity detection by TIRF microscopy. A turn-off assay is not possible since the trapping of the GFP-HA on surface antibodies is practically irreversible. We immobilized the DNA on the surface in clusters of three identical brushes, with the integrase gene expressed either from a plasmid in the bulk or from its genes immobilized in the same brush. Brushes were surrounded by surface-immobilized anti-HA antibodies arranged in a hexagonal pattern for specific detection (Figure 4A–C).

When all components of the reaction were present within the brush, including the integrase-coding gene, a fluorescent signal started appearing in the brush vicinity after a lag time of ∼20 min (15.5, 25.1, and 26.1 min across three repeats), slightly faster than a similar recombination assay performed off-chip in bulk reaction, exhibiting a lag time of ∼30 and 36 min in two separate reactions (Figure S5). Deletion of any of the assay components from the DNA brushes (promoter construct or the integrase-coding gene) reduced the fluorescent signal to the background level, allowing us to distinguish between an ON and an OFF state (Figure 4D). The localization of nascent proteins to their brush source^12^ most likely contributed to an appreciable recombination yield within the brush despite the very low concentration of integrase enzymes produced from minute amounts of immobilized integrase genes. The capture of GFP on the surface in the vicinity of the brush further contributes to the sensitivity of our turn-on assay.

Importantly, we note that the localization of the recombination and gene expression reactions to the DNA brushes enabled us to conduct and compare all control and deletion configurations in the same reaction chamber with no cross-contamination between different brush clusters (Figure 4B,C). Alternatively, the integrase could be expressed from a plasmid added to the bulk reaction above the surface to allow integrase expression to be uniform within the chamber (Figure 4E). This resulted in only a ∼15 min delay and higher rates of GFP surface accumulation closer to rates of the control construct, most likely due to higher concentration of integrase produced in the bulk.

**Figure 5:**
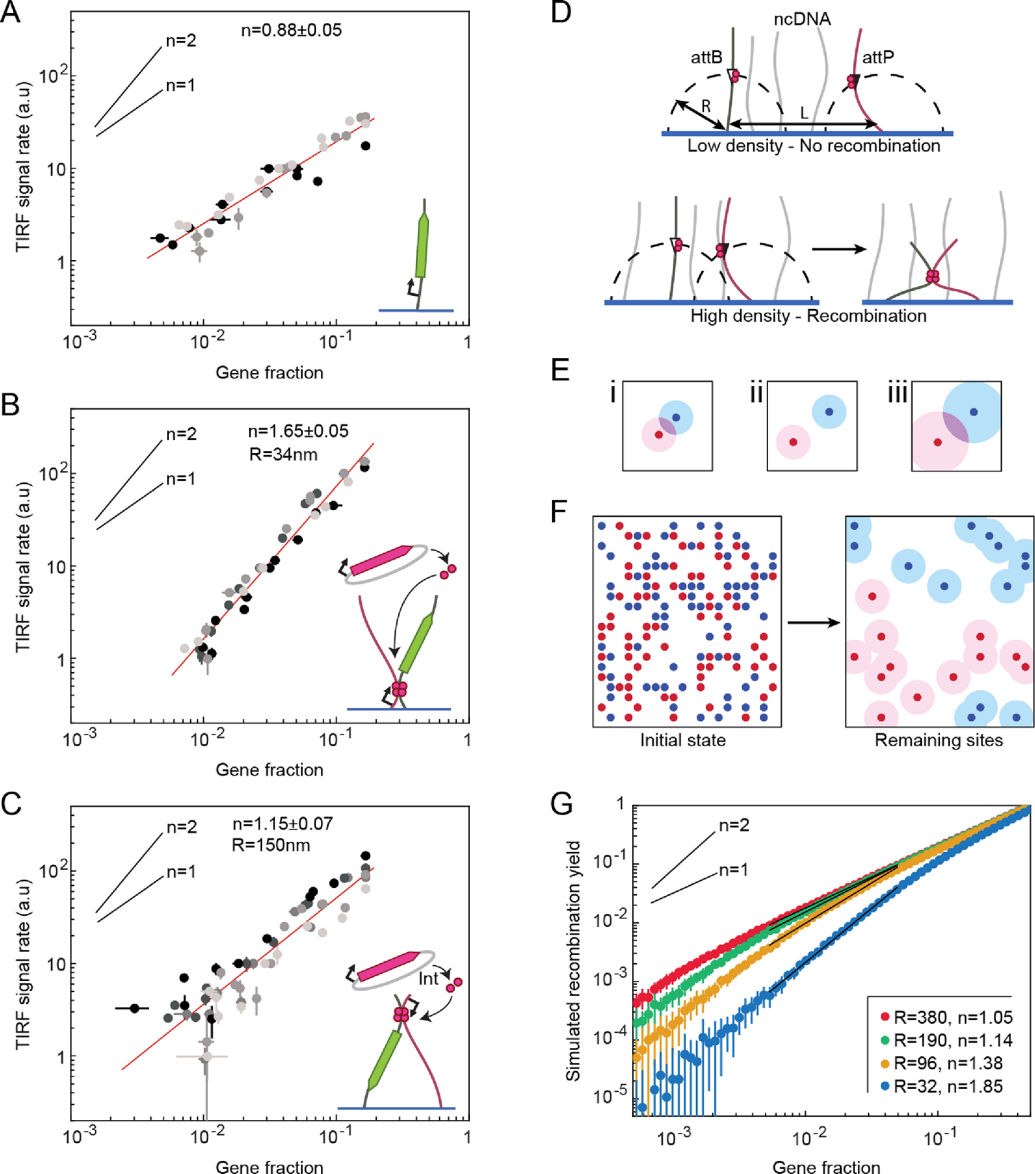
Effect of DNA site density on recombination efficiency. The accumulation rate of HA-GFP (in a.u.) as a function of gene density on a log–log scale fitted to a power law for DNA encoding HA-GFP under the T7 promoter (A), the turn-on assay with the recombination sites 100 bp from the surface (B), and 1900 bp from the surface (C). Each plot shows three to five different replicas, with points from different replicas plotted in different shades of gray. Error bars are the difference between the rates at the given timepoint and adjacent timepoints. The power laws were calculated for three repeats and averaged over them. (D) Scheme of the reach effect. Top: att sites at a distance *R* from the surface are unable to reach each other and recombine due to immobilization and low DNA density. Bottom: att sites recombine due to the overlap of the areas each of them can reach (violet area in the 2D scheme). (E, F) Reach effect simulation. The attP and attB sites are in red and blue, respectively, and recombine with each other. The probability to recombine depends on the overlap area that is high for high DNA density (i) or large *R* (iii) and low for low densities and short *R* (ii). At the end of the process, there are leftover red and blue points that cannot recombine due to their reachable areas not overlapping. (G) Simulated recombination yield in a DNA brush as a function of the fraction of each att site averaged over 25 simulations. Each curve corresponds to a different radius, as designated, that an att site is able to reach, and was fitted to a power law over a range of gene fractions.

We varied the gene fraction *D*, which effectively varies the distance between molecules, for two distances *R* between the att sites and the surface. To change the gene fraction, we diluted the DNA constructs of the GFP turn-on assay with noncoding DNA of similar length, maintaining an overall constant DNA density. Integrase was expressed from a plasmid added to the bulk (Figures 5A–C and Supporting Information). To vary the height of the sites, we used two different constructs: one in which the att sequences were positioned 1900 bp from the surface (similar to the constructs used in Figures 1–4) and one in which the construct was flipped with respect to the surface, positioning the att site 100 bp from the surface (Figures 5C and 5B, respectively). In the latter case, *R* ≈ 34 nm (using 0.34 nm/bp) since the DNA segment containing the att site was shorter than the persistence length, hence the formation of a rigid rod conformation. In the first case, the contour of the segment is a few persistence lengths, hence the formation of a relaxed coil conformation, which we estimate as *R* ≈ 150 nm,^11^ whereas for the fully stretched segment, *R* ≈ 650 nm.

The GFP fluorescence accumulation rate (the time derivative of the GFP signal) was measured at 30 min, a timepoint at which the GFP signal was restricted to the region of each DNA brush cluster with no cross-talk between clusters. The amount of fluorescently labeled DNA constructs carrying the GFP gene, proportional to the gene fraction *D*, was measured using epifluorescence (Supporting Information). We found *D* in the range of 0.007–0.125, allowing us to evaluate the average distance *L* between two molecules able to recombine: *L* = (*dD*)^−0.5^, where *d* is the brush density. Assuming *d* = 1000 DNA/μm^2^, *L* was in the range 90–380 nm.

The GFP surface accumulation rate increased with the gene fraction. We fitted the dependence of the rate on the gene fraction to a power law of order *n* over three to five repeats and found that in brushes with the sites closest to the surface, *n* = 1.65 ± 0.05, significantly different than a power of *n* = 0.88 ± 0.05 obtained in the positive control in which no recombination occurred (Figures 5,5AB and , respectively). When the recombination sites were far from the surface, we obtained a power law of *n* = 1.15 ± 0.07 (Figure 5C).

To explain these exponents, we explored two simple regimes where the sites are either dense or diluted in the brush. Consider a DNA brush made of DNA molecules of type A and B that are able to recombine with each other, each one at an equal fraction *D*, mixed with noncoding DNA. The average distance between A and B is *L*. The att sites in A and B are positioned at a distance *R* from the anchor point on the surface. In the dense case (*R* ≫ *L*), every att site is within reach of many others, so they all recombine. The total number of recombination events in the brush is then the number of A constructs, which is linear in *D*. In the dilute case (*R* ≪ *L*), most sites do not have other sites within reach and therefore are not able to recombine. In that case, the total number of recombination events corresponds to the small number of A–B pairs close enough to recombine, which is proportional to the number of A and B molecules, hence being quadratic in *D*. Between these two limits, we expect the number of recombination events to depend on *D* with a power law of exponent between 1 and 2, as experimentally measured.

To gain further insight on the measured exponents that lay between the two limits, we simulated the recombination events on a square grid of lattice step *s* = 32 nm, the average distance between DNA molecules in a brush of density *d* = 1000 DNA/μm^2^. Each grid point had a probability of *D* to be in the A and B states and 1–2*D* to be noncoding. We defined a radius *R* that these polymers were allowed to reach. A-polymers and B-polymers were allowed to recombine if the distance between them was less than 2*R*, and the total number of recombined polymers was calculated when no more polymers were able to reach each other. Figure 5G shows the recombination yield (number of recombination events divided by number of molecules in the brush) as a function of gene fraction simulated for four different *R* values chosen as integer numbers of the lattice step *s* (*R* = *s*, 3*s*, 6*s*, and 12*s*). The fit of each of these curves to a power law revealed that for all *R* values, the exponent approached 2 at low gene fractions and 1 at high gene fractions, consistent with the limit regimes.

The simulation matched quite well with the experimental data. The experimental result of *n* = 1.65 (Figure 5B) obtained for the low sites (*R* ≈ 34 nm) was closest to the value of *n* = 1.85 obtained in the simulation for *R* = 32 nm (Figure 5G). For the high sites (*R* ≈ 150 nm), we obtained an experimental result of *n* = 1.15 (Figure 5C), similar to the simulated exponent for *R* = 190 nm, consistent with our assumption that the DNA molecules were in a relaxed conformation. We conclude that the length determining recombination yield was the relaxed coil size rather than the full contour length of the DNA.

## Discussion

We have demonstrated the state-switching of gene expression in DNA-based minimal systems using recombinases. Locally expressed Bxb1 integrase showed robust compartmentalized activity, performing DNA site-specific recombination and changing the sequence of the DNA polymers in situ. Recombination between separate DNA polymers in a solution reaction had a longer lag time than in the DNA brush, indicating that the high local concentration of substrate DNA in the brush enables the integrase to perform recombination efficiently despite the low global concentration. Therefore, DNA brushes provide favorable experimental in vitro conditions to study DNA recombination processes at high density and confinement.

We showed that DNA brushes of different compositions, patterned at distances of 150 μm from each other in the same well (Figure 3), showed distinct expression kinetics reflecting different levels of recombination. This suggests that the DNA brushes compartmentalize the recombination reaction regardless of whether the integrase was encoded within the DNA brush or expressed in the bulk. Localization enables orthogonal recombination reactions within one chamber using a single recombination enzyme with no need to expand the sequence space of the recombination sites. This grants a simple way to use a single recombinase to change the regulation of several genes while still allowing the RNA and protein products to interact with each other. The recombination yield can be further controlled by physical factors such as the distance between the recombination sites and the surface as well as the ratio between the DNA substrates.

The ability to perform autonomous DNA recombination in DNA brushes by genetically encoding the integrase itself in the reaction has many possible applications, creating interesting dynamics in a cell-free brush-based protein expression system. It can be used to enlarge the sequence space of a brush or to rewire a biological circuit, changing the promoters and, hence, the activation patterns of many different genes in a single chamber based on a one-time input. This could make a certain kind of “compartmental memory” possible, in which the expression dynamics of a DNA compartment depend on previous inputs, rather than on current protein concentrations.

## Supporting Information Available

Detailed experimental procedures describing DNA preparation, preparation of linear DNA for DNA brushes, photoactivatable biocompatible monolayer assembly, assembly of large PDMS wells with DNA brushes, antibody patterning, reconstituted in vitro transcription–translation in bulk experiments, reconstituted in vitro transcription–translation on chip, integrase pre-expression experiments, total internal reflection fluorescence (TIRF) microscopy, data analysis, analysis of TIRF HA-GFP antibody trapping experiments, and simulation of recombination (PDF)
