# Supplementary material for: Toward Memory in a DNA Brush: Site-Specific Recombination Responsive to Polymer Density, Orientation, and Conformation
Source: J Am Chem Soc. 2023 Apr 18;145(17):9729–36. doi: 10.1021/jacs.3c01375 (PMC10161217; doi:10.1021/jacs.3c01375)
Supplement: Supplementary file 1 — ja3c01375_si_001.pdf [file ja3c01375_si_001.pdf]

Supplementary Information for

Toward memory in a DNA brush: site-specific recombination responsive to polymer density, orientation, and conformation

Noa Avidan[a], Michael Levy[a#], Shirley S. Daube[a]*, Roy H. Bar-Ziv[a]*

[a]Department of Chemical and Biological Physics, The Weizmann Institute of Science, Rehovot 7610001, Israel.
[#]Present address: Faculty of Biotechnology and Food Engineering, Technion, Israel Institute of Technology, Haifa 32000, Israel.

*emails: shirley.daube@weizmann.ac.il ; roy.bar-ziv@weizmann.ac.il

DNA preparations: All genes and constructs used were subcloned into pIVEX cloning vectors (Roche), which are optimized for protein expression in cell-free systems. The enhanced GFP (eGFP) F64L/S65T mutant sequence was cloned into plasmids pIVEX 2.6 under control of the T7 promoter for N-terminus hemagglutinin (HA) tagging. The Bxb1 integrase gene was amplified with the forward and reverse primers CCTCTAGAAATAATTTTGTTTAACTTTAAGAAGGAGATATACCATGAGAGCCCTGGTAGTCATCC and CGGATCTTACCGGATCTTAGTTAGTTACGACATCCCGGTGTGTAGC, respectively, from plasmid #38208 (Addgene), and inserted into pIVEX to be expressed under the T7 promoter using NEBuilder HiFi DNA assembly cloning kit (New Englands Biolabs). The attP (GTCGTGGTTTGTCTGGTCAACCACCGCGGTCTCAGTGGTGTACGGTACAAACCCCGAC) and attB (TCGGCCGGCTTGTCGACGACGGCGGTCTCCGTCGTCAGGATCATCCGGGC) sequences were inserted downstream to the T7 promoter or in the 5’ untranslated region (UTR) of the GFP gene and E. coli 16S ribosomal RNA (rRNA) genes according to Table S1. A RiboJ ribozyme sequence(1) that self cleaves the mRNA upon transcription was inserted between the att site and the ribosome binding site (RBS) of the GFP gene (Table S1) to ensure that the mRNA secondary structure is not inhibiting expression of the downstream gene. A Broccoli aptamer(2) was inserted in Helix 6 of the 16S rRNA for fluorescent labeling upon transcription, and an HDV ribozyme(3) was inserted at the 3’ end for exact cleavage of the 16S rRNA, as described in(4).

Preparation of Linear DNA for DNA Brushes: Linear double stranded DNA was amplified from pIVEX plasmids containing the relevant constructs using PCR, with the KAPA HiFi HotStart ReadyMix PCR kit (KAPA Biosystems). One primer was modified with 5' biotin, and the other was either plain or fluorescently labelled on the 5' end with ATTO-647, ATTO-488 or Alexa Fluor-647 fluorophores (Integrated DNA technologies). For all constructs, except for one, the fluorescently labeled or plain forward primer was complementary to a region 200-300 bp upstream of the T7 promoter (or the attP sequence in promoterless constructs) and the biotinylated reverse primer complementary to a region downstream to the T7 terminator. The construct with an att site positioned 100bp from the surface (Fig. 5) was prepared with a biotinylated forward primer complementary to a region 61bp upstream to the promoter and a plain reverse primer. The DNA was incubated for 5 minutes with streptavidin (Sigma) at a 1.4 SA-DNA ratio in order to create DNA-SA conjugates, and was then diluted to a final concentration of 150nM in 1x phosphate buffered saline (PBS) supplemented with 7% glycerol, the latter added to reduce evaporation at the following DNA surface deposition step.

Photoactivable Biocompatible Monolayer Assembly: Fused-silica slides (24 x 24 x 1 mm, UQG Optics) were coated with a photoactivable biocompatible monolayer according to a previously published protocol(5). Briefly, the fused-silica slides were cleaned in boiling ethanol for 10 minutes followed by sonication for 10 minutes and base piranha cleaning (H2O2:NH3:H2O; 1:1:4, heated to 70°C for 10 minutes). The slides were then coated with a polymer composed of a polyethylene glycol backbone with a protected amine at one end, and a triethoxysilyl group at the other end. The slides were incubated with the polymer solution (0.2 mg/mL in Toluene) for 20 minutes during which the monolayer is formed, and then washed and dried. The coated slides were exposed to 365 nm UV light (2.5 J/cm2) through a custom photomask containing an array of 30 μm hexagons (CAD/Art Services) using UV-KUB (Kloe). Surface amines located inside hexagons were exposed to a saturating UV dose and were fully deprotected, whereas surface amines located between hexagons were partially deprotected due to undesired leaks through the mask. In the transcription-based recombination assay the hexagonal pattern was not required

and the surface was exposed using UV-KUB without a mask. After the UV exposure the slides were immediately covered with biotin N-hydroxysuccinimidyl ester (biotin-NHS, Pierce) dissolved in a borate buffer solution (0.5 mg/mL) and incubated for 30 minutes, during which the biotin bound covalently to the exposed amine groups. The slides were then washed and dried.

Assembly of Large PDMS Wells with DNA brushes: The biotin-patterned slides were fixed on custom fused silica prisms (Zell Quarzglas und Technische Keramik) with Frame-Seal Slide Chambers adhesive (Bio-Rad). Rectangular or circular wells were cut into flat PDMS of thickness ~100 µm, and the PDMS was then applied to the biotin-patterned fused silica slide. Nano-liter DNA-SA droplets were individually deposited onto the surface inside the wells using the GIX Microplotter II (Sonoplot Inc., Middleton, WI). The droplets were incubated overnight at a humidity level of 50-65% to allow DNA brush build-up through the biotin-streptavidin linkage. For some experiments (Fig. 4, 5) antibodies were patterned on the surface surrounding the DNA brushes, as detailed below. The space between the prism and the slide was filled with index-matching liquid (Cargille) prior to imaging.

Antibody patterning: Biotinylated anti–HA antibodies (50 µg/ml; High Affinity, 3F10 clone, Sigma-Aldrich) were mixed with SA at a molar ratio of 1:1.5 in 1x PBS and incubated for 30 min on ice, followed by dilution to 50nM in 1x PBS. The solution of SA–anti-HA conjugates was used to wash the surface of the wells containing the DNA-SA droplets and incubated at 4°C for one hour. The surface was washed several times with PBS and then with 50 mM Potassium-HEPES, pH7.4, never being allowed to dry, and was kept in the HEPES buffer until the addition of the reconstituted gene expression reaction mix (PUREfrex2.0, Cosmo Bio USA).

Reconstituted in vitro transcription-translation in bulk experiments: Solutions of PUREfrex 2.0 (Cosmo Bio USA) were mixed according to the manufacturer's instructions and supplemented with 1nM of the Bxb1 integrase-encoding plasmid and 3nM of all other plasmids, as required. Reactions for measuring broccoli fluorescence were supplemented with DFHBI-1T (Lucerna, NY) at final concentrations of 60 µM.

Reconstituted in vitro transcription-translation on chip: Before the experiment, the prism containing the fused-silica slide was positioned on a temperature-controlled holder set at 17°C placed on an upright microscope (Olympus BX51WI). Humidity in the room was reduced to avoid condensation on the prism. The HEPES Potassium buffer in the chamber was exchanged for PUREfrex 2.0 (Cosmo Bio USA), as in the test-tube reactions, except that DNA was not added. Reactions in which the integrase gene was expressed from solution were supplemented with 1nM plasmids encoding the Bxb1 integrase in all experiments, except for the turn-off/turn-on experiment shown in Fig. 1 B, C in which it was at 0.25nM. The chamber was sealed with a glass coverslip, and the temperature was then switched to 37°C to trigger gene expression.

Integrase pre-expression experiments: Solutions of PUREfrex 2.0 (Cosmo Bio USA) were mixed according to the manufacturer's instructions, supplemented with 2nM plasmids expressing Bxb1 integrase and incubated off-chip at 37°C for 40 minutes. They were then mixed at a 1:1 ratio with fresh PUREfrex 2.0 solutions, containing all required plasmids and DFHBI as described above.

Total Internal Reflection Fluorescence (TIRF) Microscopy: Total Internal Reflection Fluorescence (TIRF) microscopy was used in order to image fluorescent molecules close to the surface of the FS slide inside the PDMS wells. The microscope was positioned on a motorized stage (Scientifica). It was equipped with optical filter sets for excitation at 488 and 647 nm and a fluorescent light source (EXFO X-Cite 120Q) to allow epifluorescence microscopy. Two-color TIRF microscopy was achieved by combining and coupling two lasers (OBIS 488-150 LS and OBIS 647 LX, Coherent) into a single-mode optical fiber (Oz optics). The beam was then collimated and directed on the prism using a goniometer (Thorlabs) to reach the surface of the chamber at an angle of total internal reflection, inducing an evanescent wave on the surface of the chamber. Epifluorescence and TIRF images were taken with Andor iXon Ultra camera (Andor Technology plc., Belfast, UK) and a 10X Olympus objective. The stage, the microscope, the lasers and the camera were controlled through LabVIEW (National Instruments).

Data Analysis: Images obtained from the microscope underwent basic analysis in ImageJ, used to average pixel intensity over selections in image stacks. Further analysis was done in Python and Matlab R2017b.

Analysis of TIRF HA-GFP antibody trapping experiments: Images were taken of the DNA brush clusters using the TIRF microscope, centered on the brushes themselves (see Fig. 4C). All measurements in a single experiment were done in the same chamber, at a distance of at least 1 mm from each other to prevent crosstalk. In the dilution experiments, GFP fluorescence was averaged via ImageJ over the entire image, except the central area containing the DNA brushes. The GFP fluorescence accumulation rate was calculated as the time derivative of this curve around a timepoint at which GFP signal was restricted to the region of each brush cluster (around 30 min for the recombination experiments and 7 min for the T7 GFP). In order to scale different experiments to the same range, this value was normalized by the value obtained for the 1/32 dilution (~midrange). The experimental x axis values were obtained by measuring the brush fluorescence in 647, as the DNA coding for the attP-GFP construct was fluorescently labelled in ATTO 647 and normalizing these values to fit the constructs' known dilutions via the highest dilution.

The power laws were obtained by fitting the x and y axes' logarithms to a linear function using the least squares method, and the square roots of the variance of these values are the estimated errors.

Simulation of recombination: Recombination simulations were carried out in Python 3. Each point of a 150x150 grid was randomly assigned to be a DNA molecule of type A, B, or noncoding, with predetermined probabilities. Each molecule of type A was randomly recombined with a molecule of type B within twice a given radius accessible by each molecule, and both of these molecules could no longer recombine. Molecules of type A and B that had no molecules of the other type within reach did not recombine. Recombined molecule fraction was calculated as the number of recombined A and B molecules divided by the grid size.

References

1. C. Lou, B. Stanton, Y.-J. Chen, B. Munsky, C. A. Voigt, Ribozyme-based insulator parts buffer synthetic circuits from genetic context. *Nat Biotechnol* **30**, 1137–1142 (2012).

2. G. S. Filonov, J. D. Moon, N. Svensen, S. R. Jaffrey, Broccoli: Rapid Selection of an RNA Mimic of Green Fluorescent Protein by Fluorescence-Based Selection and Directed Evolution. *J Am Chem Soc* **136**, 16299–16308 (2014).

3. S. C. Walker, J. M. Avis, G. L. Conn, General plasmids for producing RNA in vitro transcripts with homogeneous ends. *Nucleic Acids Res* **31**, e82 (2003).

4. M. Levy, R. Falkovich, S. S. Daube, R. H. Bar-ziv, Autonomous synthesis and assembly of a ribosomal subunit on a chip. *Sci Adv* **6** (2020).

5. A. Buxboim, *et al.*, A single-step photolithographic interface for cell-free gene expression and active biochips. *Small* **3**, 500–10 (2007).

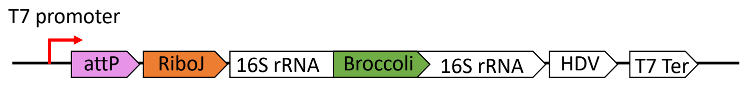 TAATACGACTCACTATAGGGAGACCACAACGGTTTCCCTCTA<br>GAGTCGTGGTTTGTCTGGTCAACCACCGCGGTCTCAGTGGT<br>GTACGGTACAAACCCCGACagctgtcaccggatgtgcTTTCCGGTC<br>TGATGAGTCCGTGAGGACGAAACAGCCTCTACAAATAATTT<br>TGTTTAAAATAAA	Broccoli-coding gene for turn-off assay and positive control.<br><br>Sequence:<br><br>Red: T7 promoter. Pink: attP. Orange: RiboJ. Green: AAA at start of 16S rRNA gene fused to the broccoli aptamer(4).
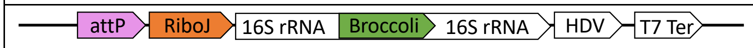 GTCGTGGTTTGTCTGGTCAACCACCGCGGTCTCAGTGGTGT<br>ACGGTACAAACCCCGACagctgtcaccggatgtgcTTTCCGGTCTG<br>ATGAGTCCGTGAGGACGAAACAGCCTCTACAAATAATTTTG<br>TTTAAAATAAA	Promoterless broccoli-coding gene for turn-on assay.<br><br>Sequence:<br><br>Pink: attP. Orange: RiboJ. Green: AAA at start of 16S rRNA gene fused to the broccoli aptamer.
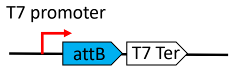 TAATACGACTCACTATAGGGAGACCACAACGGTTTCCCTCTA<br>GATCGGCCGGCTTGTCGACGACGGCGGTCTCCGTCGTCAGG<br>ATCATCCGGGCGAGCAATAACTAGCATAACCCCTTGGGGCC<br>TCTAAACGGGTCTTGAGGGGTTTTTTG	Promoter attached to attB for turn-on assay.<br><br>Sequence:<br><br>Red: T7 promoter. Light blue: attB. Violet: T7 terminator.
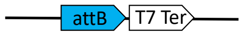 TCGGCCGGCTTGTCGACGACGGCGGTCTCCGTCGTCAGGAT<br>CATCCGGGCGAGCAATAACTAGCATAACCCCTTGGGGCCTC<br>TAAACGGGTCTTGAGGGGTTTTTTG	Promoterless attB for turn-off assay.<br><br>Sequence:<br><br>Light blue: attB. Violet: T7 terminator.
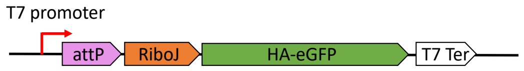 TAATACGACTCACTATAGGGAGACCACAACGGTTTCCCTCTA<br>GAGTCGTGGTTTGTCTGGTCAACCACCGCGGTCTCAGTGGT<br>GTACGGTACAAACCCCGACagctgtcaccggatgtgcTTTCCGGTC<br>TGATGAGTCCGTGAGGACGAAACAGCCTCTACAAATAATTT	HA-GFP-coding gene for positive control.<br><br>Sequence:<br><br>Red: T7 promoter. Pink: attP. Orange: RiboJ.

TGTTTAAAATAATTTTGTTTAACTTTAAGAAGGAGATATACC<br>ATG	Green: ATG at start of gene.
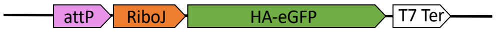 GTCGTGGTTTGTCTGGTCAACCACCGCGGTCTCAGTGGTGT<br>ACGGTACAAACCCCGACagctgtcaccggatgtgcTTTCCGGTCTG<br>ATGAGTCCGTGAGGACGAAACAGCCTCTACAAATAATTTTG<br>TTTAAAATAATTTTGTTTAACTTTAAGAAGGAGATATACCAT<br>G	Promoterless HA-GFP-coding gene for turn-on assay.<br><br>Sequence:<br><br>Pink: attP. Orange: RiboJ. Green: ATG at start of gene.

Table S1: DNA constructs.

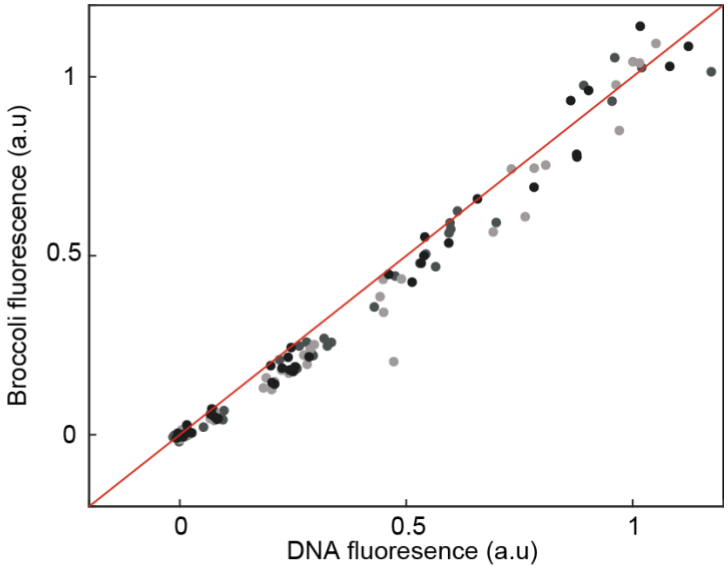

Figure S1. Broccoli signal is proportional to gene density. Broccoli signal after three minutes of expression as a function of the DNA signal of the gene-promoter construct from the experiment shown in Fig. 3. The gene-promoter construct DNA was labeled in ATTO647 dye, and the broccoli signal measured using TIRF microscopy. The broccoli signal appears to be linear in the initial amount of promoter-gene construct.

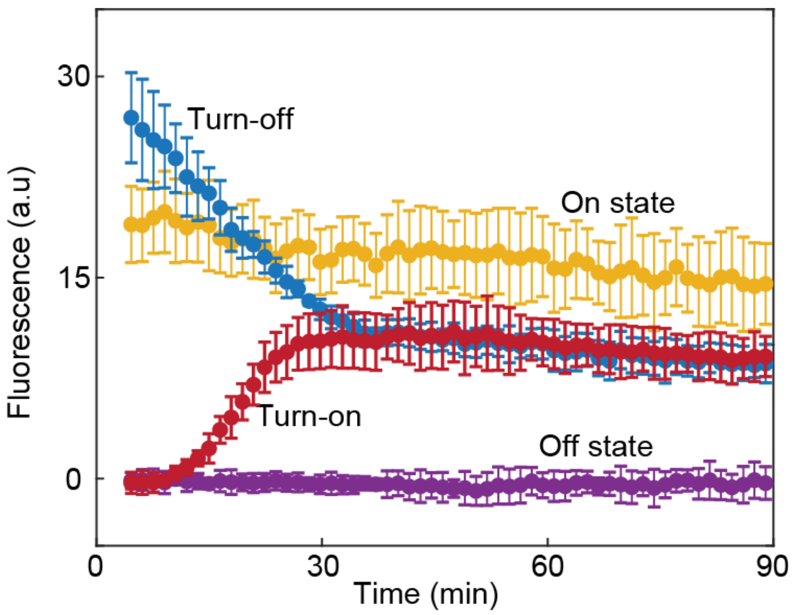

Figure S2. Raw data for figure 1 C. The kinetics of the broccoli turn-on (red) and turn-off (blue) assays, as well as a positive control consisting of broccoli constantly expressed under the T7 promoter (yellow) and a negative control consisting of promoterless broccoli gene (violet). Each signal was averaged over five repeats.

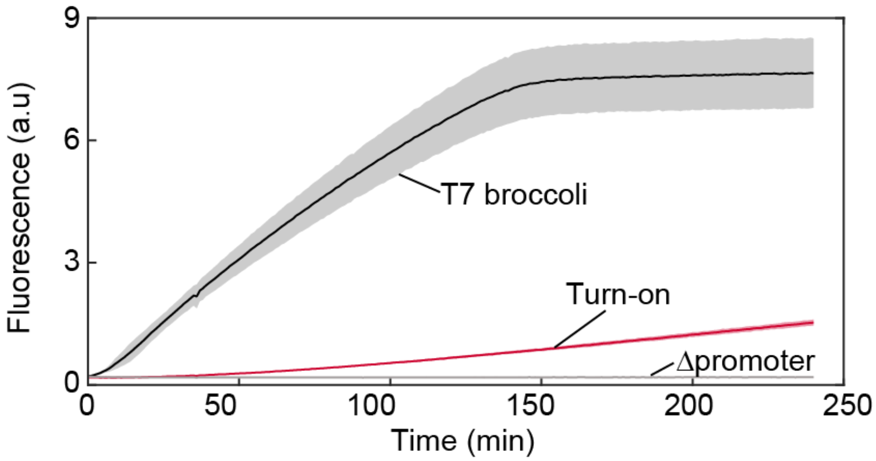

Figure S3. Recombination in solution experiment using a broccoli aptamer-based assay and pre-expressed integrase. Broccoli fluorescent signals measured in the experiment shown in Fig. 2 C, D. The kinetics of the broccoli turn-on assay as shown here were measured in the plate reader, averaged over three repeats and background subtracted.

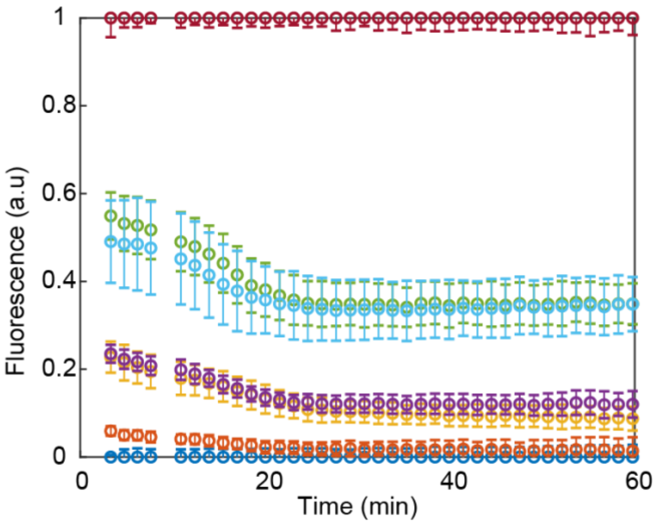

Figure S4. Recombination kinetics in efficiency experiment. Broccoli expression kinetics in the experiment shown in Fig. 3. Each curve corresponds to a different gene ratio between the gene construct and the turn-off DNA: 0:1 (blue), 1:5 (orange), 1:2 (yellow), 1:1 (violet), 2:1 (light blue), 5:1 (green) and 1:0 (red). Each curve is averaged over 15 repeats. Fluorescence signal is background-subtracted and normalized by the expression in a full brush with no recombination (dark red).

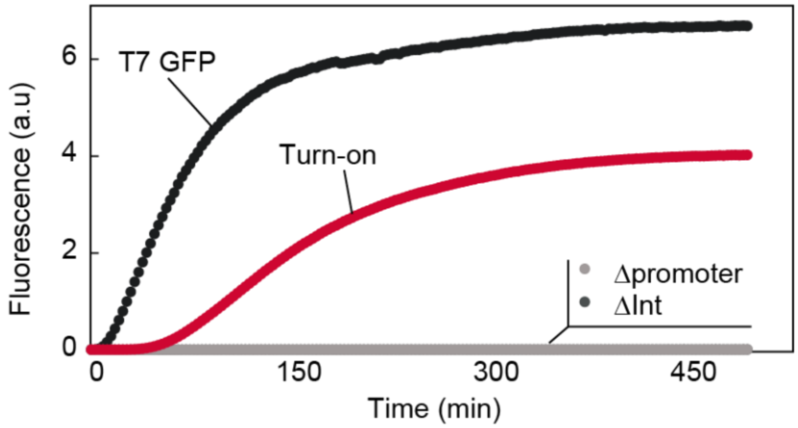

Figure S5. GFP turn-on assay solution experiment. All constructs as shown in Fig. 4 A were added as plasmids to a PUREfrex 2.0 solution reaction at 3nM, and the Bxb1 integrase gene at 1nM. The transcription-translation reactions were deposited in a V-bottom 96-well microplate (Costar) so that each well contained 5 µl of solution, and GFP fluorescence was measured every 3 minutes in a microplate reader (ClarioStar).

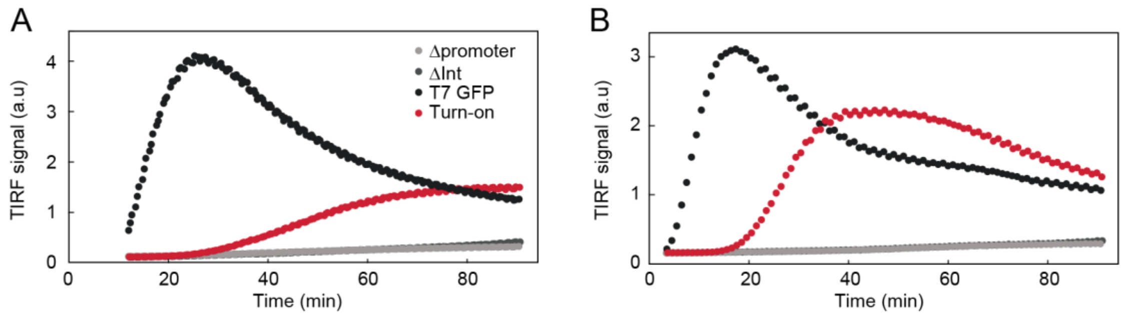

Figure S6. Repeats of the kinetic data in Fig. 4 D. HA-GFP trapping kinetics independent of recombination (black) or mediated by the integrase (red) expressed from the brush along with the promoter and HA-GFP constructs, at a ratio of 2:1:1.

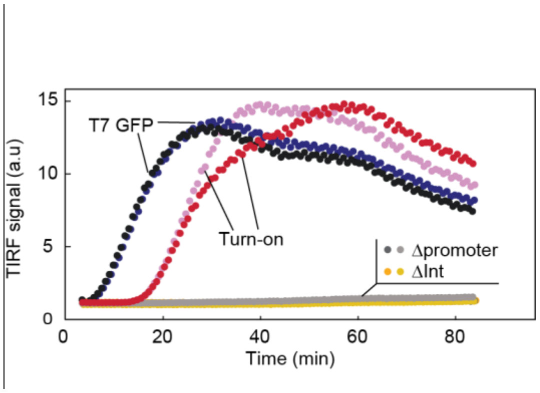

Figure S7. A single repeat of the kinetic data in Fig. 4 E. HA-GFP trapping kinetics independent of recombination (black and purple) or mediated by the integrase (red and pink). The Bxb1 integrase was expressed from a plasmid added to the bulk at a concentration of 1 nM, and the promoter and HA-GFP constructs were expressed from the DNA brush. The data shown in Fig. 4 E is presented alongside the repeated data for comparison.
